# Bipolar disorder: Trimodal age‐at‐onset distribution

**DOI:** 10.1111/bdi.13016

**Published:** 2020-11-03

**Authors:** Sorcha Bolton, Jeremy Warner, Eli Harriss, John Geddes, Kate E. A. Saunders

**Affiliations:** ^1^ Department of Psychiatry University of Oxford Warneford Hospital Oxford UK; ^2^ University of Oxford Medical School John Radcliffe Hospital Oxford UK; ^3^ Bodleian Health Care Libraries University of Oxford Oxford UK; ^4^ Oxford Health NHS Foundation Trust Warneford Hospital Oxford UK

**Keywords:** admixture analysis, age at onset, bipolar disorder, systematic review

## Abstract

**Objective:**

Bipolar disorder (BD) is a chronic mental health disorder with significant morbidity and mortality. Age at onset (AAO) may be a key variable in delineating more homogeneous subgroups of BD patients. However, no known research has systematically assessed how BD age‐at‐onset subgroups should be defined.

**Methods:**

We systematically searched the following databases: Cochrane Central Register of Controlled Trials, PsycINFO, MEDLINE, Embase, CINAHL, Scopus, Proquest Dissertations and Theses, Google Scholar and BIOSIS Previews. Original quantitative English language studies investigating AAO in BD were sought.

**Results:**

A total of 9454 unique publications were identified. Twenty‐one of these were included in data analysis (n = 22981 BD participants). Fourteen of these studies (67%, n = 13626 participants) found a trimodal AAO distribution: early‐onset (*µ* = 17.3, *σ* = 1.19, 45% of sample), mid‐onset (*µ* = 26.0, σ= 1.72, 35%), and late‐onset (*µ* = 41.9, σ= 6.16, 20%). Five studies (24%, n = 1422 participants) described a bimodal AAO distribution: early‐onset (*µ *= 24.3, *σ *= 6.57, 66% of sample) and late‐onset (*µ *= 46.3, *σ *= 14.15, 34%). Two studies investigated cohort effects on BD AAO and found that when the sample was not split by cohort, a trimodal AAO was the winning model, but when separated by cohort a bimodal distribution fit the data better.

**Conclusions:**

We propose that the field conceptualises bipolar disorder age‐at‐onset subgroups as referring broadly to life stages. Demarcating BD AAO groups can inform treatment and provide a framework for future research to continue to investigate potential mechanisms of disease onset.

## 1 INTRODUCTION

Bipolar disorder (BD) is a chronic mental health disorder with significant morbidity and mortality that affects between 1‐4% of the population.^1^ Age at onset in BD has been recognised as being important in the course and outcome of the disorder. Meta‐analytic results suggest that an early (compared to late) age at onset in bipolar disorder is associated with a longer delay to treatment, greater severity of depression and higher levels of comorbid anxiety and substance abuse.^2^, ^3^ Given this differing clinical trajectory between early‐ versus late‐onset BD, it has been proposed that age at onset (AAO) may be a key variable in delineating more homogeneous subgroups of BD patients.^4^ To date no research has systematically validated what the various AAO subgroups should be, and there is no concurrence across studies regarding what is meant by ‘early onset’.^5^, ^6^

In recent years, it has been acknowledged that AAO in bipolar disorder is not a simple unimodal distribution, but can better be explained by a mixture of distributions. Evidence has suggested that BD aggregates either into a bimodal distribution with two subgroups (early vs. late AAO), or a trimodal distribution with three subgroups (early vs. mid vs. late AAO).^3^, ^4^, ^7^, ^8^, ^9^, ^10^ However, it is not known which of these distribution modalities are more reliable and consistent. A better understanding of the distribution of age at onset in BD could provide an insight into the causes and mechanisms of illness, anticipate disease trajectory, and guide appropriate timeframes for primary and secondary prevention.^11^ Understanding the age‐at‐onset distribution of bipolar disorder over the life course also has implications relating to the conduct of clinical and epidemiological research, and health service provision and planning.

### 1.1 Objective

The aim of this systematic review was to investigate age‐at‐onset distributions in bipolar disorder, and correspondingly what constitutes an early age at onset. Only studies that use a data‐driven approach to define AAO groups were included in data synthesis, as segregating BD into AAO groups using pre‐defined cut‐offs is an inherently biased approach. One of the most popular analysis approaches for determining AAO groups is admixture analysis, as it explores the theoretical model that best fits the observed distribution of a continuous variable.

## 2 METHODS

### 2.1 Eligibility criteria

This study was pre‐registered via PROSPERO (https://bit.ly/333fs2V). All studies had to meet four criteria: 1) include participants who were recruited with a primary diagnosis of BD I, II or not otherwise specified (NOS); 2) report on the distribution of bipolar disorder AAO using a data‐driven analysis approach (e.g. admixture analysis); 3) be an original article including epidemiological, cohort, longitudinal, cross‐sectional, survey or observational studies and 4) be an English language article. Animal research, single case studies, duplicates, conference abstracts or articles with unobtainable missing data were excluded.

### 2.2 Search strategy

In February 2019 we carried out searches of the following databases: Cochrane Central Register of Controlled Trials (CENTRAL; Wiley interface), PsycINFO, MEDLINE (OVID interface, 1948 onwards), Embase (OVID interface, 1980 onwards), Cumulative Index of Nursing and Allied Health Literature (CINAHL) and Scopus. We also searched grey literature via Proquest Dissertations and Theses, BIOSIS Previews and Google Scholar.

Search strategies were developed using medical subject headings (MeSH) and text words related to bipolar disorder, age at onset and study type (Supplement S1). The syntax and subject headings of the search strategies were adapted for each database, and Boolean operators and truncation were used to extend the search terms. No date limits were imposed on the searches.

### 2.3 Study selection

The web‐based systematic review software, DistillerSR,^12^ was used to complete screening and data extraction. Two reviewers (SB, JW) independently screened titles and abstracts. A third reviewer (KS) resolved any eligibility conflicts. Following title and abstract screening, we conducted full‐text reviews of eligible articles. Where necessary, we sought additional information from study authors to resolve questions about eligibility and obtain missing data. We did not quality assess included studies as accepted standards of quality assessing non‐randomised studies are lacking,^13^ and our articles employed a broad range of study designs with diverging methodologies and reporting standards.

### 2.4 Data analysis

We extracted data from eligible studies using a standardised data extraction form. This included data on diagnoses, recruitment strategies, demographics and details of age‐at‐onset groups, including means, standard deviations (SDs) and age ranges. From our extracted data, we computed summary statistics for each study. These summary statistics related to participant characteristics (sample size, age range and gender ratio); diagnostic criteria used; age‐at‐onset definition; recruitment settings (clinic, community and hospital) and study locations. Two studies^14^, ^15^ recruited mixed samples which included participants with schizoaffective disorder; where possible, we only included participants with a BD diagnosis in analyses and samples with schizoaffective disorder participants were excluded.

We separated the studies into those reporting a trimodal AAO distribution, a bimodal distribution and those investigating cohort effects on AAO. For each study, we then extracted the average AAO per subgroup—for those studies reporting a trimodal AAO distribution we extracted the mean and SD for the early‐, mid‐ and late‐onset groups, and for those studies reporting a bimodal AAO distribution, we extracted means and SDs for the early‐ and late‐onset groups. These averages were used to plot probability density functions and boxplots for each AAO group in studies reporting a trimodal versus bimodal AAO distribution. To do this, we used the ggplot2^16^ data visualisation package in RStudio (version 1.2.1335)^17^—the analysis code can be found on our Open Science Framework (OSF) webpage (https://osf.io/5c89s/). Our data are also openly available via the OSF.^18^

## 3 RESULTS

**Figure 1:**
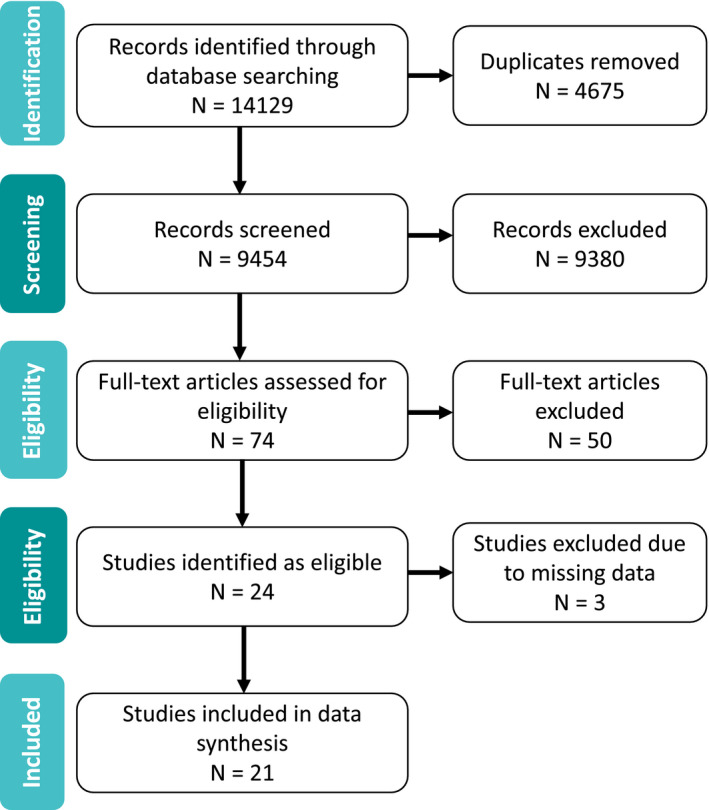
PRISMA flowchart of included studies

Our search produced 14,129 results. After duplicates were removed, we screened the titles and abstracts of 9454 articles for relevance and considered 74 eligible for full‐text review (PRISMA Diagram Figure 1; see Supplement S2 for a list of studies excluded at full‐text review). Twenty‐four articles met full‐text eligibility criteria, and 21 articles were included in data synthesis. We excluded 3 of the 24 eligible studies^19^, ^20^, ^21^ due to missing data, which were unobtainable after contacting the authors.

### 3.1 Study characteristics

All included studies were conducted from 2001 to 2017, with the majority (n = 15, 71%) published from 2009 onwards.

#### 3.1.1 Participants

Across all studies, there were a total of 22,904 bipolar disorder participants, with an average sample size of 1,094 participants per study. In total, there were 22,165 (96.78% of total) participants with a diagnosis of BDI, 653 (2.85%) with BDII, 12 (0.05%) with BD‐NOS and 74 (0.32%) with schizoaffective disorder. There were more female than male participants, with an average of 59.9% female participants across all studies.

#### 3.1.2 Age of participants at study entry

Several studies did not report age ranges or average age of their samples; those that did (n = 15) had an overall average age of 43.2 years.

#### 3.1.3 Diagnostic criteria

Thirteen studies (62%, n = 13) used DSM‐IV criteria alone to determine a bipolar disorder diagnosis.^5^, ^29^ Two studies used DSM‐IV or ICD‐10 criteria,^30^, ^31^ one used DSM‐IV or Research Diagnostic Criteria (RDC),^32^ one used DSM‐IV or DSM‐III‐R criteria,^15^ one used both DSM‐III‐R and RDC,^33^ two used RDC only^34^, ^35^ and one used case records only.^36^

#### 3.1.4 Age‐at‐onset definitions

Heterogeneous definitions of AAO were used across studies including: age at which diagnostic criteria for an affective episode was first met according to medial case notes, interviews or self‐report^5^, ^30^, ^32^, ^33^, ^34^, ^35^; age at first impairment due to an affective episode according to self‐report^8^; age at first contact with psychiatric services for symptoms of mania^14^, ^22^; age at first treatment for an affective disorder^28^ and age at first psychiatric hospitalisation.^36^ Across all studies, AAO was determined retrospectively using information gathered from medical records and/or interviews with participants and their relatives.

#### 3.1.5 Recruitment setting

Seven studies recruited patients from a clinic setting only,^9^, ^15^, ^23^, ^27^, ^29^, ^34^, ^35^ two from community settings only^8^, ^32^ and two from inpatient hospital settings only.^25^, ^36^ Three studies recruited from both the clinic and the community,^24^, ^30^, ^31^ three from both the clinic and hospital setting^5^, ^28^, ^33^ and four studies recruited from the hospital, clinic and the community.^7^, ^14^, ^22^, ^26^

#### 3.1.6 Study locations

The largest of the included studies collected data on 4037 bipolar patients across 36 collection sites in 23 countries throughout Asia, Africa, Europe, North and South America and Australia.^24^ Of the remaining studies, eleven were conducted in Europe,^5^, ^7^, ^8^, ^22^, ^23^, ^25^, ^28^, ^29^, ^31^, ^34^, ^35^ six in North America,^9^, ^14^, ^15^, ^32^, ^33^, ^36^ one in Australia^27^ and two articles combined data from collection sites in both North America and Europe.^26^, ^30^

### 3.2 Age‐at‐onset distributions

There were three separate types of distributions found for bipolar disorder age at onset across the 21 articles. Fourteen studies showed a trimodal distribution,^5^, ^32^, ^33^, ^34^, ^35^ five a bimodal distribution^9^, ^14^, ^22^, ^23^, ^36^ and two studies examined cohort effects on AAO.^24^, ^31^

#### 3.2.1 Trimodal age‐at‐onset distribution

Fourteen (67%) of the included 21 articles, including 59% (n = 13,549) of all participants, reported a trimodal age‐at‐onset distribution with three subgroups: early onset, mid‐onset and late onset (Table 1). Eight of these studies were conducted in Europe, three in America, two in both North America and Europe, and one in Australia. Of the fourteen studies, nine included participants with a diagnosis of BDI only, three with a diagnosis of BDI, BDII or BD‐NOS, and two with a diagnosis of BDI, BDII or schizoaffective disorder.

**Table 1:** Details of the studies which report a trimodal age‐at‐onset distribution in bipolar disorder

Study	N	Country	Diagnosis	Recruitment	Definition of age at onset	Method of determining AAO	Mean age of sample at study entry (SD)	Early onset		Mid‐onset		Late onset	
								Upper age limit	Mean (SD), %	Lower and upper age limits	Mean (SD), %	Lower age limit	Mean (SD), %
Azorin et al (2013)	1082	France	DSM‐IV BPI	The EPIMAN II Mille study, a multi‐centre naturalistic study conducted in 19 French medical centres	Age at which the patient first met the Research Diagnostic Criteria for an affective episode	Medical records. Structured interviews with patients and relatives.	42.9 (13.7)	20	18.6 (2.1), 19%	21‐29	24.3 (5.3), 38.9%	30	36.7 (10.8), 42%
Bellivier et al (2001)	211	France	DSM‐IV BPI	﻿Consecutive inpatients and outpatients in France	﻿Age at which DSM‐IV criteria for an affective episode was first met	Medical records. Diagnostic Interview for Genetic Studies	42.4 (14.8)		16.9 (2.7), 41.4%		26.9 (5.0), 41.8%		46.2 (8.0), 16.6%
Bellivier et al (2003)	579	France, Switzerland Germany Ireland	DSM‐IV BPI	﻿﻿Inpatients and outpatients across four countries	﻿Age at which DSM‐IV criteria for an affective episode was first met	Medical records. Diagnostic Interview for Genetic Studies	Not reported		17.4 (2.3), 27.9%		25.1 (6.2), 50.1%		40.4 (11.3), 21.9%
Bellivier et al (2014)	5891	Europe and USA	DSM‐IV BPI	Recruited for genetic, pharmacological and observational studies across 18 sites in Europe (N = 3616, incl. participants from the EMBLEM study) and from the Stanley Centre Bipolar Registry in the USA (N = 2275)	Age at which DSM‐IV criteria for an affective episode were first met	Semi‐structured interview	44.0 (13.2)	Europe					
									19 (2.7), 24.8%		27.2 (6.3), 50.7%		41.8 (10.7), 24.5%
							40.8 (11.7)	USA					
									14.5 (4.9), 63.0%		26.5 (7.6), 28.5%		39.5 (12.5), 8.5%
Biffin et al (2009)	162	Australia	DSM‐IV BPI	Recruited as part of the Bipolar Comprehensive Outcome Study (BCOS) in Melbourne, Australia	Self‐reported age at which ﻿episode of mania or depression first met diagnostic criteria	Questionnaire developed by the research team	Early:38.7 (12.6) Mid: 43.7 (12.6) Late: 58.9 (11.5)		15.5 (2.7), 44.4%		26.1 (4.8) 48.1%		50.6 (9.0), 7.4%
González‐Pinto et al (2009)	169	Spain	DSM‐IV BPI	Inpatients and outpatients who were receiving treatment in Alava, a Spanish province.	The age at first treatment for an affective disorder	Medical records. Semi‐structured SCID‐P interview. Emergency service records. Interviews with relatives.	46.0 (16.0)		18.2 (2.0), 34.0%		26.1 (5.5), 44.0%		50.9 (9.1), 22.0%
Hamshere et al (2009)	1369	UK	DSM‐IV BPI	﻿Large‐scale genetic epidemiological study. Recruited via community mental health teams, general practitioner surgeries and patient support organisations across the UK.	﻿Age at first impairment due to an affective episode according to self‐report	Medical records. Schedules for Clinical Assessment in Neuropsychiatry (SCAN).	Age range: 6 to 73 years	22	18.7 (3.7), 47.1%	25‐37	28.3 (5.5), 38.8%	40	43.3 (9.1), 14.3%
Lin et al (2006)	211	USA	DSM‐III‐R BP‐I	﻿NIMH Genetics Initiative for Bipolar Disorder ^85^	Self‐reported age at which episode of (hypo)mania or depression first met diagnostic criteria	Medical records. Diagnostic Interview for Genetic Studies.	Age range: 0 to >61 years	21	16.6 (5.1), 79.7%	22‐28	26.0 (1.4), 7.2%	28	34.7 (6.6), 13.1%
Manchia et al (2008)	181	Sardinia	RDC‐BPI	﻿Recruited from the Lithium Clinic of the Clinical Psychopharmacology Centre, University of Cagliari, Italy	﻿Age at first reliably diagnosed (hypo)manic or depressive episode	Medical records. Semi‐structured interview	42.8 (14.8)	20	18.1 (2.3), 36.0%	21‐33	24.3 (5.3), 39.0%	34	41.0 (11.5), 25.0%
﻿Nowrouzi et al (2016)	194	Canada	DSM‐III‐R or DSM‐IV BPI BPII	Recruited from four clinical sites across Ontario, Alberta and British Columbia, Canada	Unknown	Unknown	25.2 (9.51), range 14‐65 years		18.0 (2.9), 69.0%		28.7 (3.5), 22.0%		47.3 (7.8), 9.0%
Ortiz et al (2011)	379	Canada	DSM‐IV or RDC BPI BPII	﻿Recruited through the Maritime Bipolar Registry, a community‐based project in the Maritime Provinces of Canada ^86^	Age at which DSM‐IV criteria for an affective episode was first met (according to medial case notes and interviews)	Medical records. Schedule for Affective Disorders and Schizophrenia, Lifetime version	50.1 (12.7)	19	15.5 (2.0), 29.0%	20‐31	22.8 (4.6), 37.1%	32	36.1 (10.1), 33.4%
Severino et al (2009)	300	Italy	RDC BPI BPII Schizoaffective bipolar manic type	Outpatients at the Lithium Clinic of the Clinical Psychopharmacology Centre, University of Cagliari, Italy	Age at first reliably diagnosed (hypo)mania or depression according to RDC criteria (using medical records)	Medical records. Semi‐structured interview	42.9 (14.8)	22	18.5 (2.6), 43.0%	23‐37	27.5 (6.1), 42.0%	38	43.0 (10.8), 15.0%
Tozzi et al (2011)	964	UK and Canada	DSM‐IV or ICD‐10 BP‐I BP‐II	Recruited across three sites: Toronto (Canada) at the Centre for Addiction and Mental Health, London (UK), at the Institute of Psychiatry, and Dundee (UK) at the University of Dundee	Self‐reported age at which ﻿episode of mania or depression first met diagnostic criteria	Schedules for Clinical Assessment in Neuropsychiatry (SCAN) interview	47.2 (12.1), range 18‐84 years	24	16.1 (4.2), 64.0%	25	25.4 (2.5), 6.0%	26	32.2 (9.5), 30.0%
Grigoroiu‐Serbanescu et al (2014)^a^	1857	Germany Poland Romania	DSM‐IV BPI	Consecutive inpatients recruited at three sites:	Age at which DSM‐IV criteria for an affective episode were first met	Medical records. Semi‐structured interview with patients and relatives	43.4 (13.4)	Romania					
									17.6 (3.2), 43.0%		N/A	20‐21	29.9 (8.2), 57.0%
									17.3 (2.8) 33.0%		25.6 (6.3), 46.0%		40.9 (5.3), 21.0%
							44.0 (13.4)	Germany					
									20.7 (6.0), 67.0%		N/A	25	38.4 (6.5), 33.0%
									19.3 (5.5), 46.0%		28.5 (7.1), 41.0%		45.4 (4.7) 13.0%
							45.0 (14.1)	Poland					
									20.47 (3.91), 65%		N/A	24‐25	33.57 (9.12), 35%
									20.7 (3.7), 44.0%		33.0 (6.1), 45.0%		49.0 (5.3), 11.0%

Age bounds for the subgroups are provided. Numbers reported to one decimal place.

^a^ Two component and three component models fitted the data equally well.

Of the two studies including schizoaffective disorder patients,^14^, ^35^ Javaid et al (2011) report their findings including and excluding participants with schizoaffective disorder. We use the results of the ‘bipolar only’ sample in our analyses.

Two of these fourteen studies had a partial overlap in their samples^34^, ^35^ (Table 1). Manchia et al (2008) recruited 181 BDI participants from the Lithium Clinic of the Clinical Psychopharmacology Centre, University of Cagliari, Italy. Severino et al. (2009) used these same BDI participants, and additionally recruited 45 participants with BDII and 74 participants with a diagnosis of schizoaffective disorder. To account for this sample overlap and the inclusion of participants with schizoaffective disorder, we excluded these papers one by one from analyses. Excluding these studies did not make a significant difference to results (Supplement S3: Supplementary Table S1).

**Figure 2:**
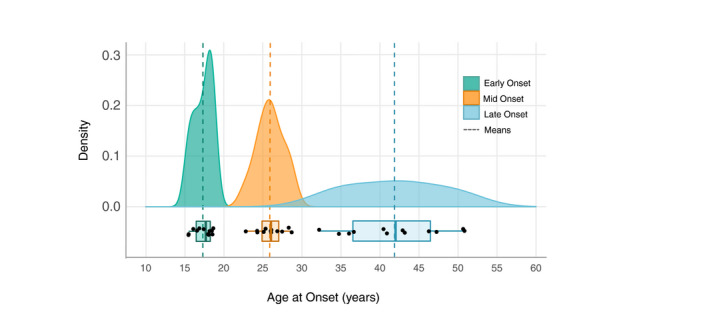
Trimodal age‐at‐onset (AAO) distribution in bipolar disorder. This figure displays the density function for each AAO group across all 14 studies, with the mean AAO per group depicted as dashed vertical lines. Under each density plot, boxplots display interquartile ranges (coloured boxes), medians (solid vertical lines) and the minima and maxima (whiskers: coloured horizontal lines)

Across these fourteen studies the average age of early, mid‐ and late onset was as follows: 17.3 years (SD = 1.19); 26.0 years (SD = 1.72) and 41.9 years (SD = 6.16). Results suggest that the majority of BD cases occurred in the early‐onset range, with an average of 45% of a total 13626 participants displaying early onset, compared to 35% mid‐onset and 20% late onset (Figure 2).

#### 3.2.2 Bimodal age‐at‐onset distribution

Five studies (24%), representing 6% (n = 1422) of all participants, described a bimodal age‐at‐onset distribution with two subgroups: early onset and late onset (Table 2). Two of these studies were conducted in Europe and three in North America. Three of the studies included participants with a diagnosis of BDI only, and two included those with a diagnosis of BDI, BDII or BD‐NOS.

**Table 2:** Details of the studies which report a bimodal age‐at‐onset distributions in bipolar disorder.

Study	N	Country	Diagnosis	Recruitment	Definition of age at onset	Method of determining AAO	Mean age of sample at study entry (SD)	Early onset		Late onset
								Upper age limit	Mean (SD), %	Mean (SD), %
Bauer et al (2010)	270	USA	DSM‐IV BPI BPII	Consecutive outpatients recruited from US clinics	Age at which ﻿episode of (hypo)mania or depression first occurred	Semi‐structured interview	Age range:≤12 to ≥30 years		15.1 (4.7), 68.1%	27.5 (10.2), 31.9%
Javaid et al (2011)	353	Canada	DSM‐IV BP or schizoaffective disorder	Recruited through newspaper advertisements and hospital clinic referrals from the Toronto region.	Age at first diagnosis of a major mood episode or mood‐related psychotic symptoms	Medical records. Structured Clinical Interview. Interviews with relatives	Whole sample: Males: 35 (10.7) Females: 36 (10.7)	22	Incl. schizoaffective disorder (n = 353)	
									16.9 (3.6)	24.4 (9.2)
									Bipolar only (n = 318)	
									16.5 (3.1)	23.7 (8.9)
Kennedy et al (2005)	246	UK	DSM‐IV BPI, first manic episode	Inpatient and outpatient cases of first‐episode mania presenting to psychiatric services in Camberwell, southeast London, between 1965 and 1999 were identified	Age at which first contact with psychiatric services was sought for mania	Medical records	Age range: 16 to ≥76	40	25.6 (6.0), 78.0%	51.0 (16.3), 22.0%
Lehmann & Rabins (2006)	73	USA	BPI	Inpatients aged >65 admitted to Johns Hopkins Hospital psychiatric service, 1990‐1995	Age at first psychiatric hospitalisation	Medical records	≥65	45	33.2 (7.4), 52.0%	64.4 (10.8), 48.0%
Manchia et al (2017)	515	Italy	DSM‐IV BPI BPII BP‐NOS	Recruited at two sites in Italy: Anxiety and Mood Disorders Unit, University of Turin, and at the Department of Psychiatry, University of Naples	Age at which DSM‐IV criteria for an affective episode was first met	Medical records. Semi‐structured interviews with patients and first‐degree relatives	47.2 (13.0)		BPI	
								32	22.6 (4.8), 67%	35.1 (10.1), 33.0%
									BPII	
								28	20.9 (4.1), 44.0%	38.2 (11.8), 56.0%
									Whole Sample	
								30	21.9 (4.6), 55.0%	37.6 (11.5), 45.0%

Age bounds for the subgroups are provided. Numbers reported to one decimal place.

**Figure 3:**
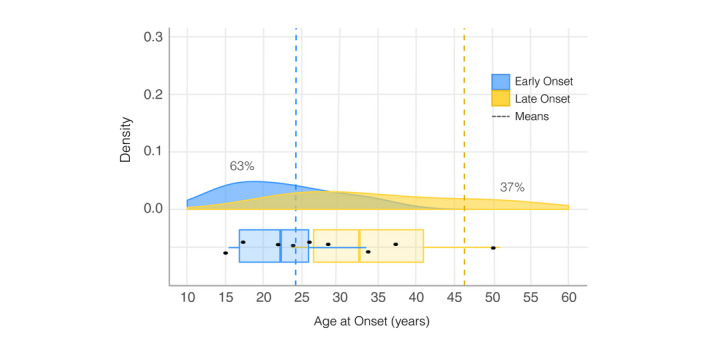
Bimodal age‐at‐onset (AAO) distribution in bipolar disorder. This figure displays the density function for each AAO group across all five studies, with the mean AAO per group depicted as dashed vertical lines. Under each density plot, boxplots display interquartile ranges (coloured boxes), medians (solid vertical lines) and the minima and maxima (whiskers: coloured horizontal lines)

Across these five studies, the average age of early onset was 22.5 years (SD = 7.32) and late onset was 40.8 years (SD = 16.89). Results indicated that an average of 63% out of a total of 1422 participants across the five studies displayed early onset, compared to 37% late onset (Figure 3).

#### 3.2.3 Effect of birth cohort

The remaining 2 of the 21 included articles examined cohort effects on AAO (Table 3). Both studies examined the effect of birth cohorts on age at onset in samples of BDI patients (total n = 7,933) recruited from clinical and community settings.

**Table 3:** Details of the studies investigating cohort effects on age‐at‐onset distributions in bipolar disorder.

Study	N	Country	Diagnosis	Recruitment	Definition of age at onset	Method of determining AAO	Mean age of sample at study entry (SD)	Cohort	Early onset	Mid‐onset	Late onset
									Mean (SD), %	Mean (SD), %	Mean (SD), %
Bauer et al. (2015)	4037	23 countries across Asia, Africa, Australia, Europe, North and South America	DSMI‐IV BPI	Data obtained retrospectively from 36 collection sites ﻿for a study of the impact of solar insolation on the age of onset of bipolar disorder	Age at first episode of depression, mania or hypomania meeting DSM‐IV criteria (according to medical case notes and interviews).	Medical records and semi‐structured interviews	48.1 (14.5)	Whole sample without birth cohorts (n = 4037) With birth cohorts incl. in model (n = 4037): born <1940, 1940‐1959, >1959 Youngest cohort, born >1959 (n = 2550)	17.2 (3.2), 41.7% 20.7 (5.8), 62.1% 18.1 (3.7), 56.9%	23.9 (5.1), 24.7% N/A N/A	32.20 (12.0), 33.6% 30.1 (10.4), 37.9% 25.8 (8.4), 43.1%
Golmard et al. (2016)	3896	Belgium, Denmark, Finland, France, Germany, Greece, Ireland, Italy, The Netherlands, Norway, Portugal, Spain, Switzerland and the UK	DSM‐IV or ICD−10 BPI	Inpatients and outpatients recruited for participation in genetic studies, and patients recruited for the EMBLEM study, a multicentre study conducted in 14 different European countries between 1993 and 2008	Age at which DSM‐IV criteria for an affective episode was first met (according to medial case notes and interviews)	Medical records. And semi‐structured clinical interviews	44.0 (13.3)	Whole sample born >1960 Whole sample born ≤1960 Matched for age at interview (n = 125): Born >1960 Born ≤1960	20.6 (3.7), 65% 19.3 (3.0), 49.7% 18.2 (2.5), 48% 16.9 (0.9), 16%	26.8 (1.7), 26% 25.9 (1.8), 32.8%	29.8 (0.5), 9% 29.8 (0.5), 17.6% 30.9 (5.3), 52% 27.1 (6.9), 84%

When the effect of birth cohort was not modelled, both studies found a trimodal bipolar disorder AAO distribution. When birth cohort was adjusted for, both studies reported that a bimodal distribution fit the data better. Across all cohorts in both studies, the overall mean ages for early, mid and late onset were 18.7 (SD = 1.52), 25.5 (SD = 1.47) and 29.4 (SD = 2.21) years, representing an average of 48.5%, 12.0% and 39.5% respectively.

#### 3.2.4 Age‐at‐onset distributions by study location and diagnostic criteria

Prior research has suggested that study location and BD diagnosis may influence AAO distributions.^37^, ^38^, ^39^, ^40^, ^41^, ^42^

##### Location

Of the eleven studies conducted in Europe, eight found a trimodal AAO distribution, two found a bimodal distribution and one reported cohort effects. There was an even split between studies reporting bi‐ and tri‐modal distributions (3 vs. 3) in North American samples. Studies conducted in both Europe and North America found a trimodal AAO distribution. The one study conducted in Australia reported a trimodal distribution.

##### Diagnosis

Two thirds of studies included participants with a diagnosis of BDI only (n = 14, 67%). Nine of these studies (64%) found a trimodal AAO distribution, compared to three reporting a bimodal distribution (21%). Five studies (25%) recruited samples with BDI, BDII and BD‐NOS. Three of these studies reported a trimodal distribution and two a bimodal distribution. Two studies included schizoaffective disorder as a diagnostic category, and both of these studies reported a trimodal AAO distribution.

The impact of diagnosis and study location does not appear to affect AAO in a meaningful way (see Supplement S3: Supplementary Table S2).

## 4 DISCUSSION

This is the first systematic review of age at onset (AAO) in bipolar disorder. The aim of this review was to provide a more reliable understanding of the AAO distribution in BD, including how ‘early‐onset’ should be defined. Our results demonstrate that a trimodal AAO distribution (early‐, mid‐ and late‐onset subgroups), compared to a bimodal distribution (early‐ vs. late‐onset), is found across a broader range of bipolar disorder diagnoses (BDI, BDII and schizoaffective disorder) and a greater number of patients (59% vs. 6% of all participants—excluding cohort studies). This provides compelling evidence to suggest that bipolar disorder onsets during early, mid or late life, with the majority (45%) of participants displaying an average age at onset of 17.3 years (SD = 1.91).

### 4.1 Defining early‐onset

Our findings offer a more robust understanding of when bipolar disorder is likely to manifest across the life course, and correspondingly provide a benchmark for what can be considered ‘early‐onset’ bipolar. We propose that a distinction should be made between ‘early‐life onset’ and ‘early‐onset’. Our results indicate that the majority of BD cases onset in early‐life, from the ages of 14‐21 years, with an average onset of 17.3 years. As it is customarily used, the term ‘early‐onset’ implies an ‘earlier than expected AAO’, whereas throughout our included studies the ‘early‐onset’ group is the most common age range for the onset of BD. We therefore recommend that the term early onset should be reconceptualised to represent life stage rather than as a comparator. ‘Early‐onset’ in the sense it is traditionally referred to is, thus, best described as onset before the age of 14 years. This distinction has the potential to aid the interpretation of existing treatment guidelines, which currently offer recommendations for treating ‘early‐onset and early‐stage’ BD without providing corresponding definitions.^43^

The diagnosis of pre‐pubertal bipolar disorder, which is prevalent in North America,^41^, ^44^ has long been viewed as contentious due to high rates of comorbidities and elevated levels of symptom overlap with other juvenile psychiatric disorders.^45^, ^46^ Our findings do not directly refute the diagnosis of paediatric bipolar disorder, but they do suggest that pre‐pubertal onset is rare. This assertion is strengthened as the included studies used samples from both Europe and North America, and is concordant with a recent meta‐analysis reporting no differences in rates of youth BD between North American and European samples.^47^ The lack of support for childhood onset may reflect the low diagnostic stability associated with very‐early‐onset BD. Evidence from longitudinal studies of high‐risk offspring suggests that manic‐like symptoms in very young children without a confirmed history of BD are not predictive of a later BD diagnosis.^48^ Additionally, epidemiological findings suggest that individuals diagnosed with BD‐NOS in childhood do go on to receive an adult BD diagnosis.^49^, ^50^ As our included studies assessed AAO retrospectively in adult samples with a confirmed BD diagnosis, any individuals who received a diagnosis of childhood BD which did not persist into adulthood will have been overlooked.

### 4.2 Treatment and diagnosis

A corollary to forming a more robust definition of ‘early‐onset’ BD is that clinical trajectory can be better anticipated, as early‐life‐onset is thought to confer a more severe and remitting course.^2^, ^3^ For instance, early‐onset BD is associated with comorbid anxiety disorders and substance abuse^2^, ^51^; clinicians should be mindful of this when assessing and treating early‐onset patients. Demarcating these AAO groups, thus, has implications for treatment provision, with the potential to guide appropriate junctures for intervention across the lifespan.

We found no substantive impact of diagnosis (BDI vs. BDII) on AAO distribution. However, most of the included studies (67%) recruited samples of BDI participants only (97% of total sample), while only 25% of studies included samples with BDII participants (3% of total sample). In light of this disparity, our findings must be interpreted with caution, especially as prior meta‐analytic research found evidence partially supporting an earlier AAO in BDI compared to BDII patients. As hypomanic episodes are not always recognised clinically, the true AAO in BDII is likely to be more difficult to determine and less reliable than BDI. Prospective follow‐up of youth at high‐risk of BD may therefore be the most reliable way to measure AAO of BDII (and/or BD‐NOS). Such longitudinal monitoring would facilitate early identification of incipient or sub‐threshold (hypo)manic and depressive symptoms.

### 4.3 Late life onset

In contrast to the finding that 45% of cases onset in the ‘early’ subgroup, only 20% of cases were deemed ‘late‐life‐onset’ (>40 years of age; Figure 2). This indicates that these two subgroups may be aetiologically distinct forms of the same disorder, as suggested by prior research.^37^, ^52^ However, late‐onset BD may have been underreported in the included studies as there was a sizeable skew towards younger samples (with an average age at study entry of 43.2 years). Additionally, a BD diagnosis in older age may be masked or missed in favour of more prevalent later‐life disorders with psychiatric symptoms (e.g. frontotemporal dementia), thus, obscuring the true rate of late‐onset BD.

### 4.4 Putative mechanisms

Our results indicate that a three‐component model (early, mid‐ and late onset) best describes the AAO distribution of bipolar disorder. As with the majority of psychiatric disorders, the interaction between genes and environment is likely to underpin the manifestation of this trimodal distribution in bipolar disorder AAO.

There is strong evidence for a genetic predisposition in bipolar disorder, with heritability estimates ranging from 60% to 85%, but the influence of genetics on AAO in bipolar is comparatively under‐studied.^53^, ^54^ Initial evidence suggests that there is genetic homogeneity within AAO subgroups and heterogeneity between groups.^55^, ^56^, ^57^ It is thought that early onset may be a more heritable form of BD than late onset, with studies demonstrating differences in transmission patterns and more pronounced familial aggregation in early‐ compared to late‐onset BD.^4^, ^6^, ^8^, ^54^, ^57^, ^58^

Genetics does not explain the whole picture, however, and there are environmental and neurobiological factors that are thought to interact with various susceptibility genes to influence the AAO of BD.^59^ It is thought that exposure to childhood trauma interacts with genes that are involved in pathways relating to neuroplasticity, inflammation and calcium signalling to influence AAO.^60^, ^61^, ^62^, ^63^ Epigenetic modifications in gene function may play an important role in the mechanism underlying the relationship between childhood trauma and a younger AAO of BD.^64^, ^65^, ^66^, ^67^, ^68^, ^69^ Childhood trauma is also associated with AAO independent of these genetic factors. Evidence suggests a dose effect of exposure to childhood trauma on the AAO of BD, with physical and sexual abuse, as well as verbal abuse, family conflict and emotional and physical neglect being significantly associated with an earlier AAO.^2^, ^51^, ^70^, ^71^, ^72^

Other candidate environmental risk factors for the subsequent onset of BD include substance abuse, decreased socioeconomic status, sleep disturbances and comorbid vascular conditions.^6^, ^73^, ^74^ Unlike childhood trauma, these factors are not unique to early life and therefore may contribute to the manifestation of mid‐ and late‐onset groups. Perhaps most relevant to the mid‐onset subgroup (onset in 20 s to early 30 s) is the phenomenon of post‐partum BD. During this time women are at increased risk for mood episodes compared with non‐postpartum periods, and childbirth has been reported as one of the most potent triggers for mania or hypomania.^75^, ^76^ It is not yet understood why childbirth is a specific trigger for manic onset, but it has been suggested that immune system dysregulation, puerperal hormones and genetic factors may activate disease pathways.^75^, ^77^ Late‐onset bipolar disorder is associated with increased rates of cerebrovascular disease, more medical and psychiatric comorbidities, and a weaker family history of psychiatric problems.^78^, ^79^ However, without employing detailed prospective longitudinal methodologies, it is unclear whether all of these environmental factors are a cause or consequence (or both) of incipient BD.

## 5 STRENGTHS AND LIMITATIONS

This is the only known systematic review investigating age at onset in bipolar disorder. To ensure we captured all relevant studies on bipolar disorder AAO, we used a search strategy with broad criteria and included several different databases and grey literature searches. We were unable to assess risk of bias due to the broad range of reporting standards and methodologies used in our included studies.

Several limitations must be considered when interpreting our findings. It has been suggested that admixture analysis is sensitive to the sample size and the characteristics of the data.^80^ The studies that reported a bimodal AAO distribution had smaller sample sizes on average compared to those reporting a trimodal AAO distribution. Interestingly, both cohort studies found a bimodal AAO distribution when they included birth cohorts in their models, but a trimodal AAO distribution when analysing the whole sample. This may be due to the fact that including birth cohorts in AAO analysis can artificially truncate the data, making the results of admixture analysis unreliable.

Inter‐study differences in findings may further be attributed to the inconsistency in the definitions used for AAO of BD, as research has suggested that admixture analysis is further sensitive to the criterion used to define groups.^80^ It has been proposed that the most valid definition for bipolar disorder AAO is the ‘first affective episode meeting diagnostic criteria’, as it does not preclude relevant episodes of depression prior to manic onset.^4^ However, this does not overcome the limitation of recall bias, which was mitigated in some of the included studies by referring to case notes and interviews with family members rather than relying solely on self‐report. Yet, BD patients may be more likely to recall depressive compared to manic episodes or even fail to recognise hypomanic episodes pre‐diagnosis as pathological.^81^, ^82^ Deciding what constitutes pathology in retrospective studies is further distorted by the fact that potential symptoms in youth are viewed through the lens of an adult diagnosis. As a gold standard, therefore, future research investigating AAO in bipolar disorder should aim to employ prospective longitudinal methodologies, using the age at ‘first affective episode meeting diagnostic criteria’ as the standardised definition for the point of disease onset.

Results will also have been influenced by factors including inter‐ and intra‐country differences in diagnostic practices, evolving diagnostic criteria, varying degrees of stigma surrounding mental illness and availability of healthcare provision. Yet, the fact that the clear majority of studies displayed a trimodal AAO despite this heterogeneity suggests that we can consider it a robust finding.

## 6 FUTURE DIRECTIONS

Notably, one of the largest international BD cohorts—the Systematic Treatment Enhancement Program for Bipolar Disorder cohort (STEP‐BD^83^)—was not included in this systematic review. This is because the STEP‐BD articles identified by our search strategy used pre‐defined cut‐offs to define AAO groups (e.g. <13, 13‐18, >18 years old) and, therefore, did not meet our eligibility criteria. The field would benefit from a data‐driven approach (such as admixture analysis) to defining BD AAO in this well‐characterised cohort. Similar analyses in other large, phenotypically detailed cohorts should also be prioritised in future research (e.g. the Flourish Canadian prospective high‐risk offspring cohort^84^).

## 7 CONCLUSION

The results of this systematic review indicate that bipolar disorder has a trimodal age‐at‐onset distribution, segregating into early‐, mid‐ and late‐onset subgroups with the most common average age at onset being 17.3 years. We propose that the field conceptualises these subgroups as referring broadly to life stage and moves towards a consistent definition of bipolar AAO as ‘the first affective episode meeting diagnostic criteria’. Providing valid evidence for three age‐at‐onset subgroups in BD will help to delineate more homogeneous subgroups of BD. Demarcating bipolar disorder AAO groups in this way can provide a framework for future research to continue to investigate potential mechanisms and, thus, inform treatment targets.

## DATA AVAILABLITY STATEMENT

The data that support the findings of this study are openly available via the Open Science Framework (OSF) at http://doi.org/10.17605/OSF.IO/XFJPR.

## Supporting information

Supplement S1

Supplement S2

Supplement S3
