# Supplementary material for: Bipolar disorder: Trimodal age‐at‐onset distribution
Source: Bipolar Disord. 2020 Nov 3;23(4):341–56. doi: 10.1111/bdi.13016 (PMC8359178; doi:10.1111/bdi.13016)
Supplement: Supplementary file 1 — Supplement S1 [file BDI-23-341-s003.docx]

Supplement 1: Search Strategy

Systematic Review search strategy in detail by database

Table of Contents

[Summary of Search Results 1](#_Toc31805858)

[MEDLINE – Ovid Interface 1](#_Toc31805859)

[Embase 2](#_Toc31805860)

[PsycINFO 3](#_Toc31805861)

[Cochrane Central Register of Controlled Trials 5](#_Toc31805862)

[CINAHL 6](#_Toc31805863)

[Scopus 9](#_Toc31805864)

[ProQuest Dissertations and Theses – Global 9](#_Toc31805865)

[BIOSIS Citation Index via Web of Science Core Collection 10](#_Toc31805866)

[Google Scholar 12](#_Toc31805867)

## Summary of Search Results

| **Database** | **Number of search results** |
| --- | --- |
| CENTRAL (searched 04/02/19) | 926 trials |
| CINAHL via EBSCO (searched 01/02/19) | 860 |
| Scopus (searched 01/02/19) | 3386 |
| ProQuest Dissertations and Theses - Global (searched 01/02/19) | 2 |
| BIOSIS Citation Index (searched 01/02/19) | 1087 |
| Ovid Medline (searched 04/02/19) | 2251 |
| Ovid Embase (searched 04/02/19) | 4325 |
| Ovid PsycINFO (searched 04/02/19) | 1292 |
| Total | 14,129 |
| Total after deduplication | 9454 |

## MEDLINE – Ovid Interface

Searched 04/02/19

Database: Medline (Ovid MEDLINE® Epub Ahead of Print, In-Process & Other Non-Indexed Citations, Ovid MEDLINE® Daily and Ovid MEDLINE®) 1946 to present

Search Strategy:

--------------------------------------------------------------------------------

1 bipolar disorder/ (38056)

2 ((bipolar or "bi polar") adj5 (disorder* or depress*)).ti,ab. (31404)

3 ((cyclothymi* or rapid or ultradian) adj5 cycl*).ti,ab. (6008)

4 (BD or BD1 or BD2 or BDi or BDii).ti,ab. (25884)

5 (hypomani* or mania* or manic* or "mixed episode*" or rcbd).ti,ab. (19488)

6 1 or 2 or 3 or 4 or 5 (79693)

7 "age of onset"/ (35352)

8 (age adj3 onset).ti,ab. (39126)

9 AAO.ti,ab. (1658)

10 ((first or initial or 1st or index or pediatric* or paediatric* or child*) adj2 (onset* or treat* or hospital* or diagnos* or symptom* or episode*)).ti,ab. (277157)

11 7 or 8 or 9 or 10 (335861)

12 6 and 11 (5146)

13 Epidemiology/ (12133)

14 exp epidemiologic studies/ (2261235)

15 observational study/ (57456)

16 epidemiolog*.ti,ab. (343434)

17 "case control".ti,ab. (113015)

18 cohort*.ti,ab. (496219)

19 "follow up stud*".ti,ab. (46369)

20 longitudinal*.ti,ab. (231040)

21 retrospective*.ti,ab. (639823)

22 "cross section*".ti,ab. (334118)

23 observational*.ti,ab. (147343)

24 ((admixture or mixture) adj3 analys*).ti,ab. (2122)

25 survey*.ti,ab. (572951)

26 13 or 14 or 15 or 16 or 17 or 18 or 19 or 20 or 21 or 22 or 23 or 24 or 25 (3586707)

27 6 and 11 and 26 (2383)

28 27 (2383)

29 limit 28 to english language (2251)

## Embase

Searched 04/02/19

Database: Embase 1974 to present

Search Strategy:

--------------------------------------------------------------------------------

1 bipolar disorder/ (47736)

2 ((bipolar or "bi polar") adj5 (disorder* or depress*)).ti,ab. (47508)

3 ((cyclothymi* or rapid or ultradian) adj5 cycl*).ti,ab. (7535)

4 (BD or BD1 or BD2 or BDi or BDii).ti,ab. (45251)

5 (hypomani* or mania* or manic* or "mixed episode*" or rcbd).ti,ab. (26379)

6 1 or 2 or 3 or 4 or 5 (117727)

7 onset age/ (75599)

8 (age adj3 onset).ti,ab. (62544)

9 AAO.ti,ab. (1989)

10 ((first or initial or 1st or index or pediatric* or paediatric* or child*) adj2 (onset* or treat* or hospital* or diagnos* or symptom* or episode*)).ti,ab. (430619)

11 7 or 8 or 9 or 10 (523962)

12 exp epidemiology/ (3043419)

13 epidemiolog*.ti,ab. (429254)

14 "case control".ti,ab. (145828)

15 cohort*.ti,ab. (830828)

16 "follow up stud*".ti,ab. (58958)

17 longitudinal*.ti,ab. (305473)

18 retrospective*.ti,ab. (1046991)

19 "cross section*".ti,ab. (422696)

20 observational*.ti,ab. (229646)

21 ((admixture or mixture) adj3 analys*).ti,ab. (2453)

22 survey*.ti,ab. (723157)

23 observational study/ (159721)

24 12 or 13 or 14 or 15 or 16 or 17 or 18 or 19 or 20 or 21 or 22 or 23 (5292815)

25 6 and 11 and 24 (4545)

26 25 (4545)

27 limit 26 to english language (4325)

## PsycINFO

Searched 04/02/19

Database: PsycINFO 1806 to present

Search Strategy:

--------------------------------------------------------------------------------

1 exp bipolar disorder/ (25506)

2 ((bipolar or "bi polar") adj5 (disorder* or depress*)).ti,ab. (30151)

3 ((cyclothymi* or rapid or ultradian) adj5 cycl*).ti,ab. (2453)

4 (BD or BD1 or BD2 or BDi or BDii).ti,ab. (11700)

5 (hypomani* or mania* or manic* or "mixed episode*" or rcbd).ti,ab. (20752)

6 1 or 2 or 3 or 4 or 5 (51786)

7 "onset (disorders)"/ (12142)

8 (age adj3 onset).ti,ab. (13291)

9 AAO.ti,ab. (171)

10 ((first or initial or 1st or index or pediatric* or paediatric* or child*) adj2 (onset* or treat* or hospital* or diagnos* or symptom* or episode*)).ti,ab. (59200)

11 7 or 8 or 9 or 10 (77912)

12 exp epidemiology/ (47714)

13 epidemiolog*.ti,ab. (43138)

14 "case control".ti,ab. (9961)

15 cohort*.ti,ab. (68739)

16 "follow up stud*".ti,ab. (12043)

17 longitudinal*.ti,ab. (105777)

18 retrospective*.ti,ab. (39544)

19 "cross section*".ti,ab. (71755)

20 observational*.ti,ab. (24642)

21 ((admixture or mixture) adj3 analys*).ti,ab. (428)

22 survey*.ti,ab. (273755)

23 12 or 13 or 14 or 15 or 16 or 17 or 18 or 19 or 20 or 21 or 22 (566394)

24 6 and 11 and 23 (1408)

25 24 (1408)

26 limit 25 to english language (1292)

## Cochrane Central Register of Controlled Trials

Searched 04/02/19

Issue 2 of 12, February 2019

Date Run: 04/02/2019 17:28:20

#1 MeSH descriptor: [Bipolar Disorder] explode all trees (2326)

#2 ((bipolar or "bi polar") near/5 (disorder* or depress*)) (5248)

#3 ((cyclothymi* or rapid or ultradian) near/5 cycl*) (377)

#4 BD or BD1 or BD2 or BDi or BDii (8125)

#5 hypomani* or mania* or manic* or "mixed episode*" or rcbd (3033)

#6 #1 or #2 or #3 or #4 or #5 (13997)

#7 MeSH descriptor: [Age of Onset] explode all trees (599)

#8 age near/3 onset (2269)

#9 AAO (268)

#10 ((first or initial or 1st or index or pediatric* or paediatric* or child*) near/2 (onset* or treat* or

hospital* or diagnos* or symptom* or episode*)) (54719)

#11 #7 or #8 or #9 or #10 (56649)

#12 #6 and #11 (1346)

= 926 trials

## CINAHL

Searched 01/02/19

Friday, February 01, 2019 12:55:31 PM Top of Form

| # | Query | Limiters/Expanders | Last Run Via | Results |
| --- | --- | --- | --- | --- |
| S26 | S6 AND S11 AND S24 | Narrow by Language: - english  Search modes - Boolean/Phrase | Interface - EBSCOhost Research Databases  Search Screen - Advanced Search  Database - CINAHL | 860 |
| S25 | S6 AND S11 AND S24 | Search modes - Boolean/Phrase | Interface - EBSCOhost Research Databases  Search Screen - Advanced Search  Database - CINAHL | 868 |
| S24 | S12 OR S13 OR S14 OR S15 OR S16 OR S17 OR S18 OR S19 OR S20 OR S21 OR S22 OR S23 | Search modes - Boolean/Phrase | Interface - EBSCOhost Research Databases  Search Screen - Advanced Search  Database - CINAHL | 1,080,269 |
| S23 | TI survey* OR AB survey* | Search modes - Boolean/Phrase | Interface - EBSCOhost Research Databases  Search Screen - Advanced Search  Database - CINAHL | 211,381 |
| S22 | TI ( ((admixture or mixture) adj3 analys* ) OR AB ( ((admixture or mixture) adj3 analys* ) | Search modes - Boolean/Phrase | Interface - EBSCOhost Research Databases  Search Screen - Advanced Search  Database - CINAHL | 0 |
| S21 | TI observational* OR AB observational* | Search modes - Boolean/Phrase | Interface - EBSCOhost Research Databases  Search Screen - Advanced Search  Database - CINAHL | 53,233 |
| S20 | TI "cross section*" OR AB "cross section*" | Search modes - Boolean/Phrase | Interface - EBSCOhost Research Databases  Search Screen - Advanced Search  Database - CINAHL | 113,142 |
| S19 | TI retrospective* OR AB retrospective* | Search modes - Boolean/Phrase | Interface - EBSCOhost Research Databases  Search Screen - Advanced Search  Database - CINAHL | 154,943 |
| S18 | TI longitudinal* OR AB longitudinal* | Search modes - Boolean/Phrase | Interface - EBSCOhost Research Databases  Search Screen - Advanced Search  Database - CINAHL | 71,682 |
| S17 | TI "follow up stud*" OR AB "follow up stud*" | Search modes - Boolean/Phrase | Interface - EBSCOhost Research Databases  Search Screen - Advanced Search  Database - CINAHL | 9,852 |
| S16 | TI cohort* OR AB cohort* | Search modes - Boolean/Phrase | Interface - EBSCOhost Research Databases  Search Screen - Advanced Search  Database - CINAHL | 165,411 |
| S15 | TI "case control" OR AB "case control" | Search modes - Boolean/Phrase | Interface - EBSCOhost Research Databases  Search Screen - Advanced Search  Database - CINAHL | 27,199 |
| S14 | TI epidemiolog* OR AB epidemiolog* | Search modes - Boolean/Phrase | Interface - EBSCOhost Research Databases  Search Screen - Advanced Search  Database - CINAHL | 62,438 |
| S13 | (MH "Epidemiological Research+") | Search modes - Boolean/Phrase | Interface - EBSCOhost Research Databases  Search Screen - Advanced Search  Database - CINAHL | 27,303 |
| S12 | (MH "Epidemiology+") | Search modes - Boolean/Phrase | Interface - EBSCOhost Research Databases  Search Screen - Advanced Search  Database - CINAHL | 588,665 |
| S11 | S7 OR S8 OR S9 OR S10 | Search modes - Boolean/Phrase | Interface - EBSCOhost Research Databases  Search Screen - Advanced Search  Database - CINAHL | 103,930 |
| S10 | TI ( ((first or initial or 1st or index or pediatric* or paediatric* or child*) n2 (onset* or treat* or hospital* or diagnos* or symptom* or episode*)) ) OR AB ( ((first or initial or 1st or index or pediatric* or paediatric* or child*) n2 (onset* or treat* or hospital* or diagnos* or symptom* or episode*)) ) | Search modes - Boolean/Phrase | Interface - EBSCOhost Research Databases  Search Screen - Advanced Search  Database - CINAHL | 90,217 |
| S9 | TI AAO OR AB AAO | Search modes - Boolean/Phrase | Interface - EBSCOhost Research Databases  Search Screen - Advanced Search  Database - CINAHL | 649 |
| S8 | TI age n3 onset OR AB age n3 onset | Search modes - Boolean/Phrase | Interface - EBSCOhost Research Databases  Search Screen - Advanced Search  Database - CINAHL | 7,407 |
| S7 | (MH "Age of Onset") | Search modes - Boolean/Phrase | Interface - EBSCOhost Research Databases  Search Screen - Advanced Search  Database - CINAHL | 9,981 |
| S6 | S1 OR S2 OR S3 OR S4 OR S5 | Search modes - Boolean/Phrase | Interface - EBSCOhost Research Databases  Search Screen - Advanced Search  Database - CINAHL | 17,699 |
| S5 | TI ( hypomani* or mania* or manic* or "mixed episode*" or rcbd ) OR AB ( hypomani* or mania* or manic* or "mixed episode*" or rcbd ) | Search modes - Boolean/Phrase | Interface - EBSCOhost Research Databases  Search Screen - Advanced Search  Database - CINAHL | 3,284 |
| S4 | TI ( BD or BD1 or BD2 or BDi or BDii ) OR AB ( BD or BD1 or BD2 or BDi or BDii ) | Search modes - Boolean/Phrase | Interface - EBSCOhost Research Databases  Search Screen - Advanced Search  Database - CINAHL | 4,838 |
| S3 | TI ( ((cyclothymi* or rapid or ultradian) n5 cycl*) ) OR AB ( ((cyclothymi* or rapid or ultradian) n5 cycl*) ) | Search modes - Boolean/Phrase | Interface - EBSCOhost Research Databases  Search Screen - Advanced Search  Database - CINAHL | 511 |
| S2 | TI ( ((bipolar or "bi polar") n5 (disorder* or depress*)) ) OR AB ( ((bipolar or "bi polar") n5 (disorder* or depress*)) ) | Search modes - Boolean/Phrase | Interface - EBSCOhost Research Databases  Search Screen - Advanced Search  Database - CINAHL | 8,871 |
| S1 | (MH "Bipolar Disorder+") | Search modes - Boolean/Phrase | Interface - EBSCOhost Research Databases  Search Screen - Advanced Search  Database - CINAHL | 10,050 |

## Scopus

Searched 01/02/19

( ( TITLE-ABS-KEY ( ( ( bipolar OR "bi polar" ) W/5 ( disorder* OR depress* ) ) ) ) OR ( TITLE-ABS-KEY ( ( ( cyclothymi* OR rapid OR ultradian ) W/5 cycl* ) ) ) OR ( TITLE-ABS-KEY ( bd OR bd1 OR bd2 OR bdi OR bdii ) ) OR ( TITLE-ABS-KEY ( ( hypomani* OR mania* OR manic* OR "mixed episode*" OR rcbd ) ) ) ) AND ( ( TITLE-ABS-KEY ( age W/3 onset ) ) OR ( TITLE-ABS-KEY ( aao ) ) OR ( TITLE-ABS-KEY ( ( ( first OR initial OR 1st OR index OR pediatric* OR paediatric* OR child* ) W/2 ( onset* OR treat* OR hospital* OR diagnos* OR symptom* OR episode* ) ) ) ) ) AND ( ( TITLE-ABS-KEY ( epidemiolog* OR "case control" OR cohort* OR "follow up stud*" OR longitudinal* OR retrospective* OR "cross section*" OR observational* OR survey* ) ) OR ( TITLE-ABS-KEY ( ( ( admixture OR mixture ) W/3 analys* ) ) ) ) AND ( LIMIT-TO ( LANGUAGE , "English" ) )

## ProQuest Dissertations and Theses – Global

Searched 01/02/19

((ti((bipolar OR "bi polar") NEAR/5 (disorder* OR depress*)) OR ab((bipolar OR "bi polar") NEAR/5 (disorder* OR depress*))) OR (ti((cyclothymi* OR rapid OR ultradian) NEAR/5 cycl*) OR ab((cyclothymi* OR rapid OR ultradian) NEAR/5 cycl*)) OR (ti(bd OR bd1 OR bd2 OR bdi OR bdii ) OR ab(bd OR bd1 OR bd2 OR bdi OR bdii )) OR (ti(hypomani* OR mania* OR manic* OR "mixed episode*" OR rcbd) OR ab(hypomani* OR mania* OR manic* OR "mixed episode*" OR rcbd))) AND (ti(( admixture OR mixture ) near/3 analys* ) OR ab(( admixture OR mixture ) near/3 analys* )) AND ((ti(epidemiolog* OR "case control" OR cohort* OR "follow up stud*" OR longitudinal* OR retrospective* OR "cross section*" OR observational* OR survey*) OR ab(epidemiolog* OR "case control" OR cohort* OR "follow up stud*" OR longitudinal* OR retrospective* OR "cross section*" OR observational* OR survey*)) OR (ti(( admixture OR mixture ) near/3 analys* ) OR ab(( admixture OR mixture ) near/3 analys* )))

## BIOSIS Citation Index via Web of Science Core Collection

Searched 01/02/19

| # 1 | TOPIC: (( bipolar OR "bi polar" ) near/5 ( disorder* OR depress* ))  Indexes=BCI Timespan=All years | 28,534 |
| --- | --- | --- |
| # 2 | TOPIC: ((cyclothymi* OR rapid OR ultradian ) near/5 cycl*)  Indexes=BCI Timespan=All years | 6,169 |
| # 3 | TOPIC: (bd OR bd1 OR bd2 OR bdi OR bdii)  Indexes=BCI Timespan=All years | 19,963 |
| # 4 | TOPIC: (hypomani* OR mania* OR manic* OR "mixed episode*" OR rcbd)  Indexes=BCI Timespan=All years | 18,432 |
| # 5 | #4 OR #3 OR #2 OR #1  Indexes=BCI Timespan=All years | 61,740 |
| # 6 | TOPIC: (age near/3 onset)  Indexes=BCI Timespan=All years | 29,646 |
| # 7 | TOPIC: (AAO)  Indexes=BCI Timespan=All years | 815 |
| # 8 | TOPIC: (( first OR initial OR 1st OR index OR pediatric* OR paediatric* OR child* ) near/2 ( onset* OR treat* OR hospital* OR diagnos* OR symptom* OR episode* ))  Indexes=BCI Timespan=All years | 206,624 |
| # 9 | #8 OR #7 OR #6  Indexes=BCI Timespan=All years | 233,226 |
| # 10 | TOPIC: (epidemiolog* OR "case control" OR cohort* OR "follow up stud*" OR longitudinal* OR retrospective* OR "cross section*" OR observational* OR survey*)  Indexes=BCI Timespan=All years | 1,955,702 |
| # 11 | TOPIC: (( admixture OR mixture ) near/3 analys*)  Indexes=BCI Timespan=All years | 5,570 |
| # 12 | #11 OR #10  Indexes=BCI Timespan=All years | 1,960,951 |
| # 13 | #12 AND #9 AND #5  Indexes=BCI Timespan=All years | 1,113 |
| # 14 | #12 AND #9 AND #5  Refined by: LANGUAGES: ( ENGLISH )  Indexes=BCI Timespan=All years | 1,087 |

## Google Scholar

Screened the first 10 pages of results for each of the following (sorted by relevance):

("bipolar disorder"|"bi polar disorder"|"bipolar depress*"|"bi polar depress*"|"cyclothymi* cycl*"|"rapid cycl*"|"ultradian cycl*"|bd|bd1|bd2|bdi|bdii|hypomani*|mania*|manic*|"mixed episode*"|rcbd)("age of onset"|"onset age"|AAO|"first diagno*")

<https://scholar.google.co.uk/scholar?hl=en&as_sdt=0%2C5&q=%28%22bipolar+disorder%22%7C%22bi+polar+disorder%22%7C%22bipolar+depress*%22%7C%22bi+polar+depress*%22%7C%22cyclothymi*+cycl*%22%7C%22rapid+cycl*%22%7C%22ultradian+cycl*%22%7Cbd%7Cbd1%7Cbd2%7Cbdi%7Cbdii%7Chypomani*%7Cmania*%7Cmanic*%7C%22mixed+episode*%22%7Crcbd%29%28%22age+of+onset%22%7C%22onset+age%22%7CAAO%7C%22first+diagno*%22%29&btnG>

("bipolar disorder"|"bi polar disorder"|"bipolar depress*"|"bi polar depress*"|"cyclothymi* cycl*"|"rapid cycl*"|"ultradian cycl*"|bd|bd1|bd2|bdi|bdii|hypomani*|mania*|manic*|"mixed episode*"|rcbd)("first symptom*"|"first epidsode*"|"first onset*"|"first treat*"|"first hospital*)

<https://scholar.google.co.uk/scholar?hl=en&as_sdt=0%2C5&q=%28%22bipolar+disorder%22%7C%22bi+polar+disorder%22%7C%22bipolar+depress*%22%7C%22bi+polar+depress*%22%7C%22cyclothymi*+cycl*%22%7C%22rapid+cycl*%22%7C%22ultradian+cycl*%22%7Cbd%7Cbd1%7Cbd2%7Cbdi%7Cbdii%7Chypomani*%7Cmania*%7Cmanic*%7C%22mixed+episode*%22%7Crcbd%29%28%22first+symptom*%22%7C%22first+epidsode*%22%7C%22first+onset*%22%7C%22first+treat*%22%7C%22first+hospital*%29&btnG>

("bipolar disorder"|"bi polar disorder"|"bipolar depress*"|"bi polar depress*"|"cyclothymi* cycl*"|"rapid cycl*"|"ultradian cycl*"|bd|bd1|bd2|bdi|bdii|hypomani*|mania*|manic*|"mixed episode*"|rcbd)("initial onset*"|"initial treat*"|"initial hospital*")

<https://scholar.google.co.uk/scholar?hl=en&as_sdt=0%2C5&q=(%22bipolar+disorder%22%7C%22bi+polar+disorder%22%7C%22bipolar+depress*%22%7C%22bi+polar+depress*%22%7C%22cyclothymi*+cycl*%22%7C%22rapid+cycl*%22%7C%22ultradian+cycl*%22%7Cbd%7Cbd1%7Cbd2%7Cbdi%7Cbdii%7Chypomani*%7Cmania*%7Cmanic*%7C%22mixed+episode*%22%7Crcbd)(%22initial+onset*%22%7C%22initial+treat*%22%7C%22initial+hospital*%22)&btnG>

("bipolar disorder"|"bi polar disorder"|"bipolar depress*"|"bi polar depress*"|"cyclothymi* cycl*"|"rapid cycl*"|"ultradian cycl*"|bd|bd1|bd2|bdi|bdii|hypomani*|mania*|manic*|"mixed episode*"|rcbd)("initial diagnos*"|"initial symptom*"|"initial episode*")

<https://scholar.google.co.uk/scholar?hl=en&as_sdt=0%2C5&q=%28%22bipolar+disorder%22%7C%22bi+polar+disorder%22%7C%22bipolar+depress*%22%7C%22bi+polar+depress*%22%7C%22cyclothymi*+cycl*%22%7C%22rapid+cycl*%22%7C%22ultradian+cycl*%22%7Cbd%7Cbd1%7Cbd2%7Cbdi%7Cbdii%7Chypomani*%7Cmania*%7Cmanic*%7C%22mixed+episode*%22%7Crcbd%29%28%22initial+diagnos*%22%7C%22initial+symptom*%22%7C%22initial+episode*%22%29&btnG>
