# Supplementary material for: Bipolar disorder: Trimodal age‐at‐onset distribution
Source: Bipolar Disord. 2020 Nov 3;23(4):341–56. doi: 10.1111/bdi.13016 (PMC8359178; doi:10.1111/bdi.13016)
Supplement: Supplementary file 2 — Supplement S2 [file BDI-23-341-s002.docx]

Supplement 2

Papers excluded at full-text review

1. Alda, M. (2013). Does age at onset define a subtype of bipolar disorder? *Bipolar Disorders*, *15*(SUPPL.1), 28. https://doi.org/http://dx.doi.org/10.1111/bdi.12079
2. Alda, M., Grof, P., Ravindran, L., Cavazzoni, P., Duffy, A., Grof, E., … Wilson, J. (2000). Anticipation in bipolar affective disorder: is age at onset a valid criterion?. *American Journal of Medical Genetics*, *96*(6), 804–807.
3. Azorin, J.-M., Belzeaux, R., & Adida, M. (2015). Age-at-onset and comorbidity may separate depressive disorder subtypes along a descending gradient of bipolar propensity. *Behavioural Brain Research*, *282*, 185–193. https://doi.org/https://dx.doi.org/10.1016/j.bbr.2015.01.014
4. Baldessarini, R. J., Bolzani, L., Cruz, N., Jones, P. B., Lai, M., Lepri, B., … Vieta, E. (2010). Onset-age of bipolar disorders at six international sites. *Journal of Affective Disorders*, *121*(1–2), 143–146. https://doi.org/https://dx.doi.org/10.1016/j.jad.2009.05.030
5. Bauer, M. (2012). Factors associated with age at onset of bipolar disorder. *Bipolar Disorders*, *14*(SUPPL. 1), 33–34. https://doi.org/http://dx.doi.org/10.1111/j.1399-5618.2012.00977.x
6. Bauer, M., Glenn, T., Alda, M., Aleksandrovich, M. A., Andreassen, O. A., Angelopoulos, E., … Whybrow, P. C. (2017). Solar insolation in springtime influences age of onset of bipolar I disorder. *Acta Psychiatrica Scandinavica*, *136*(6), 571–582. https://doi.org/https://dx.doi.org/10.1111/acps.12772
7. Bellivier, F. (n.d.). Time trends of age at onset of bipolar I disorder. *Bipolar Disorders*, *15*(SUPPL.1), 28–29. https://doi.org/http://dx.doi.org/10.1111/bdi.12079
8. Benazzi, F. (1998). Late-life depression in private practice depressed outpatients: a 203-case study. *International Journal of Geriatric Psychiatry*, *13*(3), 145–148.
9. Benazzi, F. (2009). Classifying mood disorders by age-at-onset instead of polarity. *Progress in Neuro-Psychopharmacology and Biological Psychiatry*, *33*(1), 86–93. https://doi.org/10.1016/j.pnpbp.2008.10.007
10. Benazzi, Franco. (2007). Does age at onset support a dimensional relationship between Bipolar II disorder and major depressive disorder?. *The World Journal of Biological Psychiatry : The Official Journal of the World Federation of Societies of Biological Psychiatry*, *8*(2), 105–111.
11. Bogren, M., Mattisson, C., Isberg, P.-E., Munk-Jorgensen, P., & Nettelbladt, P. (2010). Incidence of psychotic disorders in the 50 year follow up of the Lundby population. *Australian and New Zealand Journal of Psychiatry*, *44*(1), 31–39. https://doi.org/http://dx.doi.org/10.3109/00048670903393647
12. Burke, K. C., Burke, J. D. J., Rae, D. S., & Regier, D. A. (1991). Comparing age at onset of major depression and other psychiatric disorders by birth cohorts in five US community populations. *Archives of General Psychiatry*, *48*(9), 789–795.
13. Burke, K. C., Burke, J. D. J., Regier, D. A., & Rae, D. S. (1990). Age at onset of selected mental disorders in five community populations. *Archives of General Psychiatry*, *47*(6), 511–518.
14. Chengappa, K. N. R., Kupfer, D. J., Frank, E., Houck, P. R., Grochocinski, V. J., Cluss, P. A., & Stapf, D. A. (2003). Relationship of birth cohort and early age at onset of illness in a bipolar disorder case registry. *The American Journal of Psychiatry*, *160*(9), 1636–1642.
15. Coryell, W., Fiedorowicz, J., Leon, A. C., Endicott, J., & Keller, M. B. (2013). Age of onset and the prospectively observed course of illness in bipolar disorder. *Journal of Affective Disorders*, *146*(1), 34–38. https://doi.org/https://dx.doi.org/10.1016/j.jad.2012.08.031
16. Da Silva Magalhães, P. V, Gomes, F. A., Kunz, M., & Kapczinski, F. (2009). Birth-cohort and dual diagnosis effects on age-at-onset in Brazilian patients with bipolar i disorder. *Acta Psychiatrica Scandinavica*, *120*(6), 492–495. https://doi.org/10.1111/j.1600-0447.2009.01426.x
17. Dell’Osso, B., Buoli, M., Riundi, R., D’Urso, N., Pozzoli, S., Bassetti, R., … Altamura, A. C. (2009). Clinical characteristics and long-term response to mood stabilizers in patients with bipolar disorder and different age at onset. *Neuropsychiatric Disease and Treatment*, *5*, 399–404.
18. De Luca, V. (2015). Age at onset in first episode bipolar disorder: Effect of recall bias and cultural influences. *Bipolar Disorders*, *17*(SUPPL. 1), 121. https://doi.org/http://dx.doi.org/10.1111/bdi.12309
19. Depp, C. A., Jin, H., Mohamed, S., Kaskow, J., Moore, D. J., & Jeste, D. V. (2004). Bipolar disorder in middle-aged and elderly adults: is age of onset important?. *The Journal of Nervous and Mental Disease*, *192*(11), 796–799.
20. Dodd, S., Ng, F., Berk, M., Fitzgerald, P., de Castella, A., Kulkarni, J., … Montgomery, B. (2008). Early age of onset is associated with high rates of anxiety comorbiditity in bipolar I and schizoaffective disorders. *International Journal of Neuropsychopharmacology*, *11*(Suppl. 1), 179.
21. Fogarty, F., Russell, J. M., Newman, S. C., & Bland, R. C. (1994). Epidemiology of psychiatric disorders in Edmonton: Phenomenology and comorbidity: Mania. *Acta Psychiatrica Scandinavica*, *89*(SUPPL. 376), 16–23. https://doi.org/10.1111/j.1600-0447.1994.tb05787.x
22. Goldberg, J. F., & Garno, J. L. (2009). Age at onset of bipolar disorder and risk for comorbid borderline personality disorder. *Bipolar Disorders*, *11*(2), 205–208. https://doi.org/10.1111/j.1399-5618.2008.00653.x
23. Golmard, J.-L., Etain, B., Scott, J., Malafosse, A., Henry, C., Jamain, S., … Bellivier, F. (2011). Birth Cohort and Period Effects on Age at Onset in Bipolar I Affective Disorder. *Biological Psychiatry*, *69*(9, Suppl. S), 55S.
24. Golmard, J.-L., Scott, J., Etain, B., Malafosse, A., Henry, C., Jamain, S., … Bellivier, F. (2013). Birth Cohort Effect on Age at Onset of Bipolar I Disorder : Are We Entering the Age of Mania? *Biological Psychiatry*, *73*(9, Suppl. S), 171S.
25. Grande, I., Magalhaes, P. V, Chendo, I., Stertz, L., Panizutti, B., Colpo, G. D., … Vieta, E. (2014). Staging bipolar disorder: clinical, biochemical, and functional correlates. *Acta Psychiatrica Scandinavica*, *129*(6), 437–444. https://doi.org/https://dx.doi.org/10.1111/acps.12268
26. Grigoroiu-Serbanescu, M., Nothen, M. M., Ohlraun, S., Propping, P., Maier, W., Wickramaratne, P., … Rietschel, M. (2005). Family history influences age of onset in bipolar I disorder in females but not in males. *American Journal of Medical Genetics. Part B, Neuropsychiatric Genetics : The Official Publication of the International Society of Psychiatric Genetics*, *133B*(1), 6–11.
27. Haouala, B., Amamou, B., Allegue, M., & Zaafrane, F. (2016). Age at onset of bipolar disorders: Clinical implication and prognosis of early and late onset. *European Neuropsychopharmacology*, *26*(Supplement 2), S429.
28. Holtzman, J. N., Miller, S., Hooshmand, F., Wang, P. W., Chang, K. D., Hill, S. J., … Ketter, T. A. (2015). Childhood-compared to adolescent-onset bipolar disorder has more statistically significant clinical correlates. *Journal of Affective Disorders*, *179*, 114–120. https://doi.org/10.1016/j.jad.2015.03.019
29. Kennedy, N., Boydell, J., Kalidindi, S., Fearon, P., Jones, P. B., van Os, J., & Murray, R. M. (2005). Gender differences in incidence and age at onset of mania and bipolar disorder over a 35-year period in Camberwell, England. *The American Journal of Psychiatry*, *162*(2), 257–262.
30. Kroon, J. S., Wohlfarth, T. D., Dieleman, J., Sutterland, A. L., Storosum, J. G., Denys, D., & de Haan, L. (2013). Incidence rates and risk factors of bipolar disorder in the general population: A population-based cohort study. *Bipolar Disorders*, *15*(3), 306–313. https://doi.org/http://dx.doi.org/10.1111/bdi.12058
31. Larsson, S., Lorentzen, S., Mork, E., Barrett, E. A., Steen, N. E., Lagerberg, T. V., … Andreassen, O. A. (2010). Age at onset of bipolar disorder in a Norwegian catchment area sample. *Journal of Affective Disorders*, *124*(1–2), 174–177. https://doi.org/https://dx.doi.org/10.1016/j.jad.2009.10.031
32. Manchia, M., Lampus, S. F., Margiani, S., Chillotti, C., Ardau, R., Severino, G., & Del Zompo, M. (2006). Bipolar disorder in Sardinian population: Age at onset as an endophenotype? *American Journal of Medical Genetics*, *141B*(7), 776.
33. Marchand, W., Wirth, L., & Simon, C. (2004). Delayed diagnosis of pediatric bipolar disorder in a community sample. *Journal of Affective Disorders*, *78*(Supplement 1), S60–S61.
34. Masi, G., Perugi, G., Millepiedi, S., Mucci, M., Toni, C., Bertini, N., … Pari, C. (2006). Developmental differences according to age at onset in juvenile bipolar disorder. *Journal of Child and Adolescent Psychopharmacology*, *16*(6), 679–685.
35. Matteo, P., & Stefano, P. (2015). Age at onset in a sample of bipolar patients: clinical features and psychiatric comorbidity. *European Neuropsychopharmacology*, *25*(Suppl. 2), S388. https://doi.org/10.1016/s0924-977x(15)30507-1
36. Mérette, C., Roy-Gagnon, M. H., Ghazzali, N., Savard, F., Boutin, P., Roy, M. A., & Maziade, M. (2000). Anticipation in schizophrenia and bipolar disorder controlling for an information bias. *American Journal of Medical Genetics - Neuropsychiatric Genetics*, *96*(1), 61–68. https://doi.org/10.1002/(SICI)1096-8628(20000207)96:1<61::AID-AJMG13>3.0.CO;2-W
37. Moorhead, S. R. J., & Young, A. H. (2003). Evidence for a late onset bipolar-I disorder sub-group from 50 years. *Journal of Affective Disorders*, *73*(3), 271–277.
38. Negash, A., Alem, A., Kebede, D., Deyessa, N., Shibre, T., & Kullgren, G. (2005). Prevalence and clinical characteristics of bipolar I disorder in Butajira, Ethiopia: a community-based study. *Journal of Affective Disorders*, *87*(2–3), 193–201.
39. Oedegaard, K. J., Syrstad, V. E. G., Morken, G., Akiskal, H. S., & Fasmer, O. B. (2009). A study of age at onset and affective temperaments in a Norwegian sample of patients with mood disorders. *Journal of Affective Disorders*, *118*(1–3), 229–233. https://doi.org/https://dx.doi.org/10.1016/j.jad.2009.01.030
40. Peselow, E. D., Dunner, D. L., Fieve, R. R., Deutsch, S. I., & Rubinstein, M. E. (1982). Age of onset of affective illness. *Psychiatria Clinica*, *15*(3), 124–132.
41. Post, R. M., Altshuler, L. L., Kupka, R., McElroy, S. L., Frye, M. A., Rowe, M., … Nolen, W. A. (2016). Age of onset of bipolar disorder: Combined effect of childhood adversity and familial loading of psychiatric disorders. *Journal of Psychiatric Research*, *81*, 63–70. https://doi.org/https://dx.doi.org/10.1016/j.jpsychires.2016.06.008
42. Post, R. M., & Kowatch, R. A. (2006). The health care crisis of childhood-onset bipolar illness: some recommendations for its amelioration. *The Journal of Clinical Psychiatry*, *67*(1), 115–125.
43. Post, R. M., Kupka, R., Keck, P. E. J., McElroy, S., Altshuler, L., Frye, M., … Nolen, W. (2015). Evidence for a cohort effect: A child network for assessing naturalistic treatment in the community. *Bipolar Disorders*, *17*(SUPPL. 1), 106. https://doi.org/http://dx.doi.org/10.1111/bdi.12309
44. Roy-Byrne, P., Post, R. M., Uhde, T. W., Porcu, T., & Davis, D. (1985). THE LONGITUDINAL COURSE OF RECURRENT AFFECTIVE ILLNESS LIFE CHART DATA FROM RESEARCH PATIENTS AT THE NATIONAL INSTITUTE OF MENTAL HEALTH. *Acta Psychiatrica Scandinavica Supplementum*, *71*(317), 1–34. https://doi.org/10.1111/j.1600-0447.1985.tb10510.x
45. Scott, J., Etain, B., Azorin, J. M., & Bellivier, F. (2018). Secular trends in the age at onset of bipolar I disorder - Support for birth cohort effects from interational, multi-centre clinical observational studies. *European Psychiatry : The Journal of the Association of European Psychiatrists*, *52*, 61–67. https://doi.org/https://dx.doi.org/10.1016/j.eurpsy.2018.04.002
46. Singh, N., MacMohan, H., Bilderbeck, A., Reed, Z. E., Tunbridge, E., & Churchill, G. C. (2016). Plasma glutathione as a marker of oxidative stress in bipolar disorder. *European Neuropsychopharmacology*, *26*(Supplement 2), S421–S422.
47. Smeraldi, E., Gasperini, M., Macciardi, F., Bussoleni, C., & Morabito, A. (n.d.). Factors affecting the distribution of age at onset in patients with affective disorders. *Journal of Psychiatric Research*, *17*(3), 309–317.
48. Smeraldi, E., Macciardi, F., Holmgren, S., Perris, H., von Knorring, L., & Perris, C. (1987). Age at onset of affective disorders in Italian and Swedish patients. *Acta Psychiatrica Scandinavica*, *75*(4), 352–357.
49. Tillman, R., Geller, B., Bolhofner, K., Craney, J. L., Williams, M., & Zimerman, B. (2003). Ages of onset and rates of syndromal and subsyndromal comorbid DSM-IV diagnoses in a prepubertal and early adolescent bipolar disorder phenotype. *Journal of the American Academy of Child and Adolescent Psychiatry*, *42*(12), 1486–1493.
50. Vaingankar, J. A., Rekhi, G., Subramaniam, M., Abdin, E., & Chong, S. A. (2013). Age of onset of life-time mental disorders and treatment contact. *Social Psychiatry and Psychiatric Epidemiology*, *48*(5), 835–843. https://doi.org/https://dx.doi.org/10.1007/s00127-012-0601-y
