# Supplementary material for: Bipolar disorder: Trimodal age‐at‐onset distribution
Source: Bipolar Disord. 2020 Nov 3;23(4):341–56. doi: 10.1111/bdi.13016 (PMC8359178; doi:10.1111/bdi.13016)
Supplement: Supplementary file 3 — Supplement S3 [file BDI-23-341-s001.docx]

Supplement 3

# Overlapping Samples: Trimodal Age at Onset Distribution

Manchia et al. (2008) and Severino et al. (2009) both use an overlapping sample of 181 BPI participants recruited from the Lithium Clinic of the Clinical Psychopharmacology Centre, University of Cagliari, Italy. The later 2009 Severino et al. paper additionally includes 74 participants with a diagnosis of schizoaffective disorder and 45 participants with BPII.

We have repeated our analysis excluding first the Manchia et al. (2008) paper, and then the Severino et al. (2009) paper. This does not make a significant difference to the overall means (and SDs) per AAO group, or to the proportion of participants in each AAO group:

**Supplementary Table 1.** Descriptive stats excluding Manchia et al. (2008) and Severino et al. (2009).

|  | **Currently reported in paper** | | **Excluding Manchia et al. (2008)** | | **Excluding Severino et al. (2009)** | |
| --- | --- | --- | --- | --- | --- | --- |
|  | Mean (SD) | Proportion of pps per group | Mean (SD) | Proportion of pps per group | Mean (SD) | Proportion of pps per group |
| **Early onset** | 17.34 (1.19) | 44.54% | 17.27 (1.22) | 45.32% | 17.23 (1.89) | 44.68% |
| **Mid onset** | 25.96 (1.73) | 34.51% | 26.11 (1.72) | 34.10% | 25.82 (1.74) | 33.83% |
| **Late onset** | 41.87 (6.16) | 20.81% | 41.95 (6.45) | 20.43% | 41.76 (6.45) | 21.33% |

Additionally, removing these studies did not make a substantial difference when plotting our final model, as can be seen from the following three figures.

As the exclusion of the papers does not significantly alter our findings and interpretation of the data, we have chosen to include both studies.

1. As currently reported. Not excluding Manchia et al. (2008) or Severino et al. (2009)


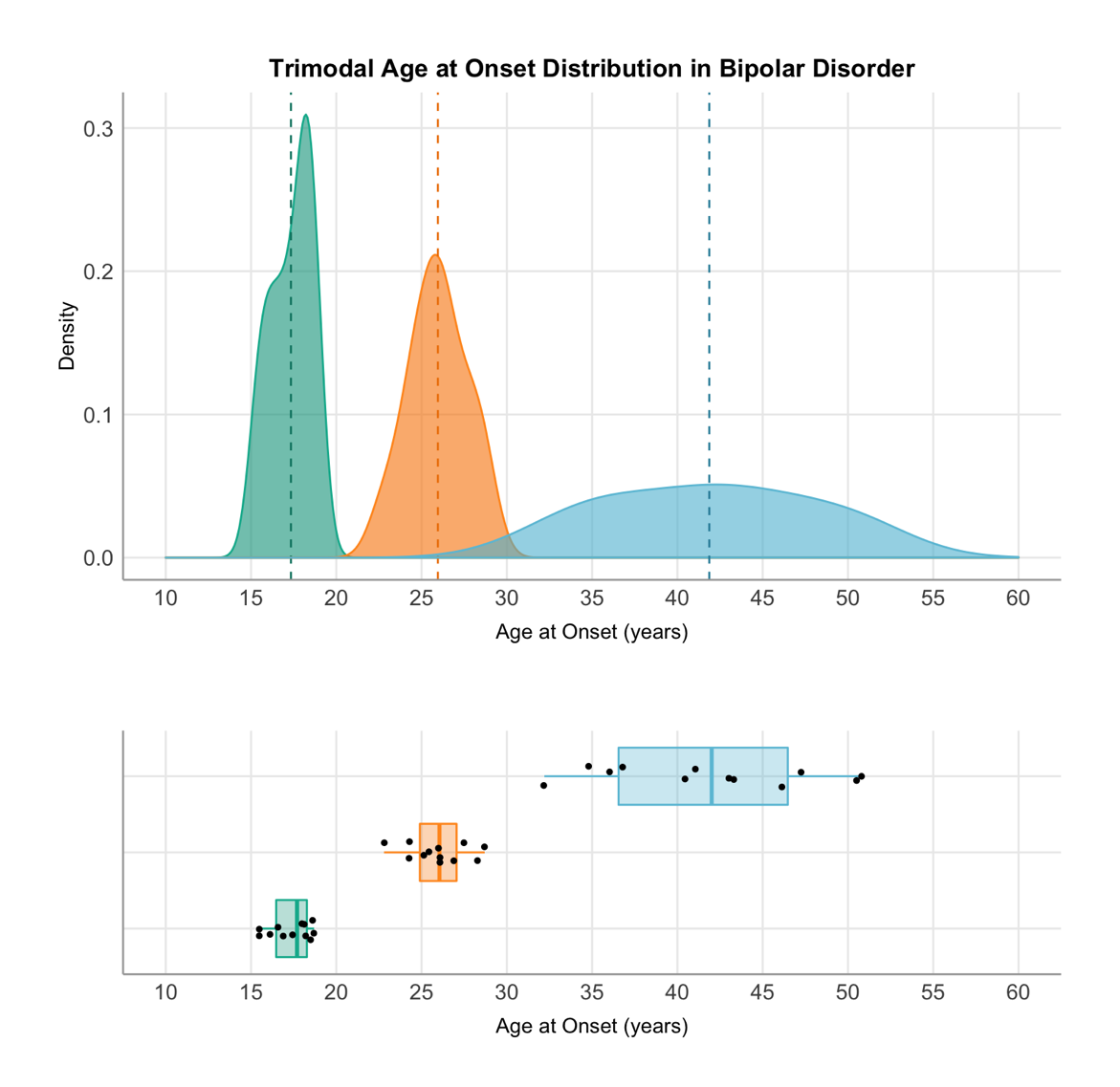

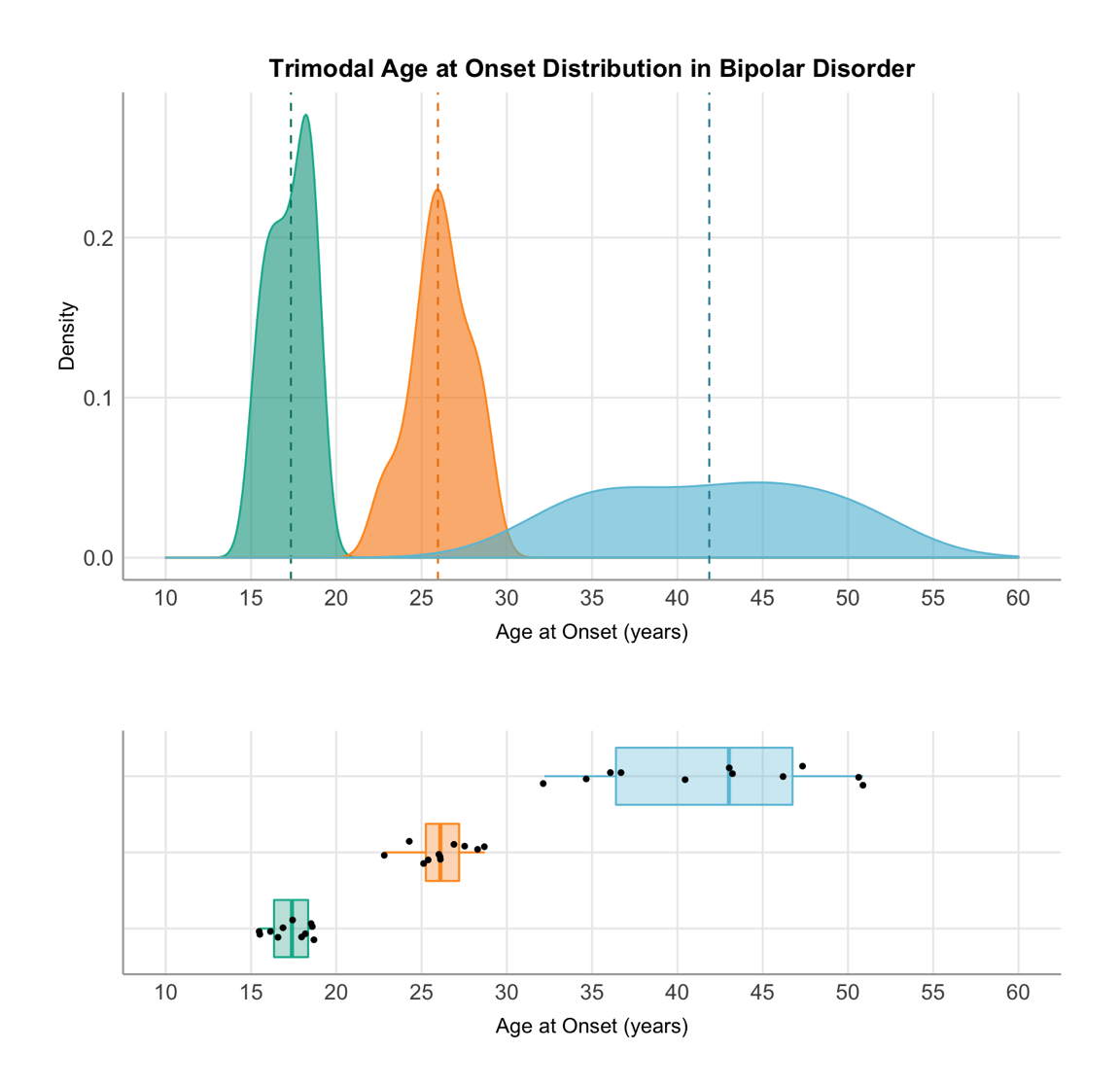


1. Excluding Manchia et al. (2008)


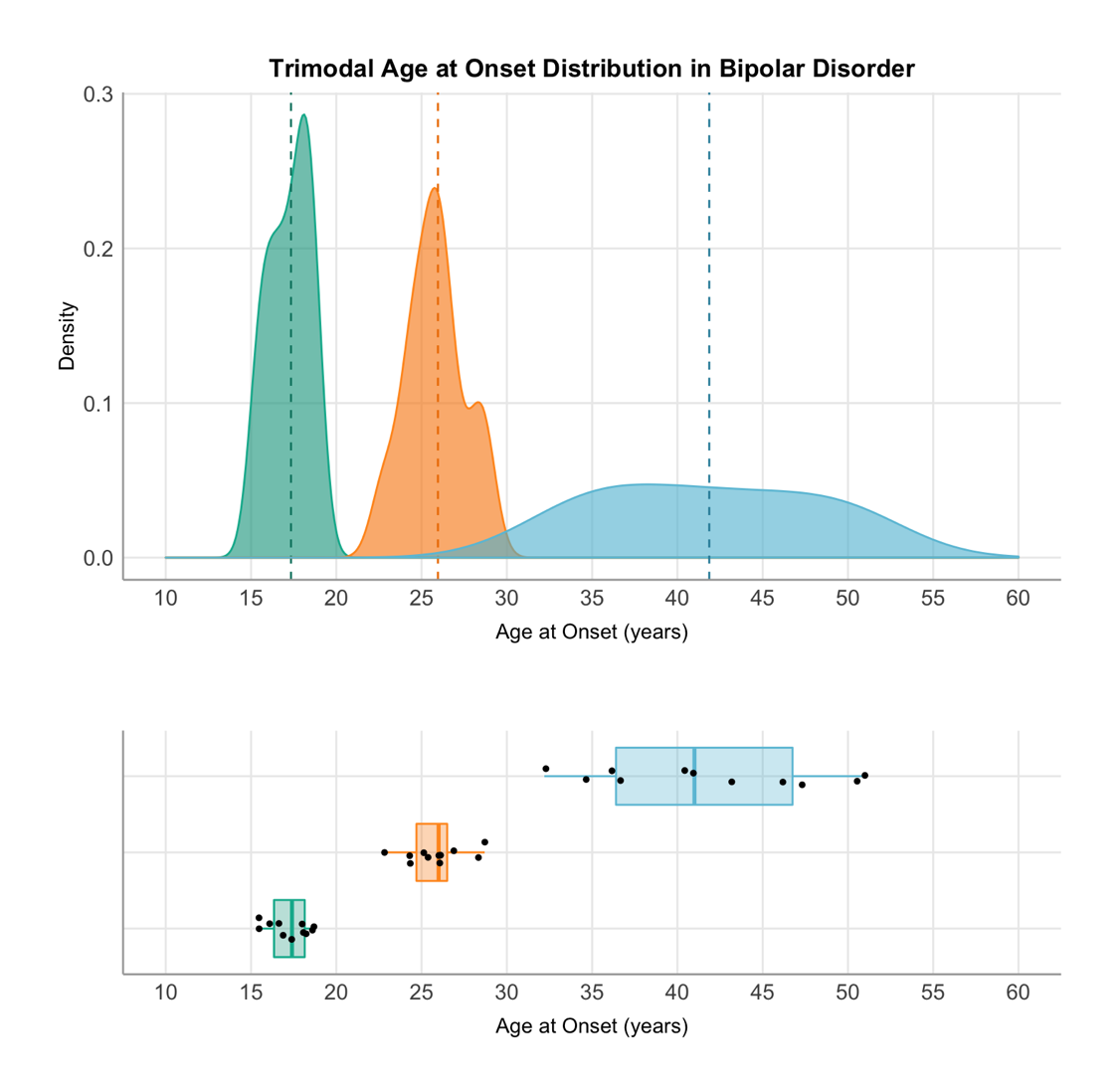


1. Excluding Severino et al. (2009)

# Age at onset distributions by study location and diagnostic criteria

**Supplementary Table 2.** Age-at-onset distributions according to location in which the study was conducted and diagnostic category.

| **Location** | **Number of studies** | **AAO Distributions** | | |
| --- | --- | --- | --- | --- |
|  |  | **Bimodal** | **Trimodal** | **Birth Cohort** |
| Europe | 11 | 2 | 8 | 1 |
| North America | 6 | 3 | 3 | 0 |
| Australia | 1 | 0 | 1 | 0 |
| Europe and North America | 2 | 0 | 2 | 0 |
| Worldwide | 1 | 0 | 0 | 1 |
| Total | 21 | 5 | 14 | 2 |
| **Diagnostic Category** |  |  |  |  |
| BPI | 14 | 3 | 9 | 2 |
| BPI and BPII | 5 | 2 | 3 | 0 |
| BPI, BPII and Schizoaffective Disorder | 2 | 0 | 2 | 0 |
| Total | 21 | 5 | 14 | 2 |

We carried out the Freeman-Halton extension of the Fisher exact probability test to examine whether study location or diagnostic category impacted on the reported AAO distribution (bimodal or trimodal). Both of these were non-significant:

- Location: two-tailed Fisher’s exact test, P = 0.497
- Diagnosis: two-tailed fishers exact test, P = 0.598
